# Decreased trabecular bone score in patients affected by Fabry disease

**DOI:** 10.1007/s40618-024-02427-x

**Published:** 2024-10-03

**Authors:** Emanuele Varaldo, Beatrice Giannone, Francesca Viglino, Fabio Settanni, Fabio Bioletto, Marco Barale, Massimo Procopio, Silvia Deaglio, Ezio Ghigo, Andrea Benso

**Affiliations:** 1 Division of Endocrinology, Diabetology and Metabolism, Department of Medical Sciences, University of Turin, Corso Dogliotti, 14, Turin, 10126 Italy; 2 Endocrine Unit, Diabetes Regional Center, Treviglio, Italy; 3 Division of Clinical Biochemistry, Department of Laboratory Medicine, University of Turin, Turin, Italy; 4 Oncological Endocrinology Unit, Department of Oncology, University of Turin, Turin, Italy; 5 Immunogenetics and Transplant Biology Unit, Department of Medical Sciences, University of Turin, Turin, Italy

**Keywords:** TBS, Bone quality, Bone microarchitecture, Inborn errors of metabolism, FD, Osteoporosis, Osteopenia

## Abstract

**Background:**

Fabry disease (FD) is an inherited X-linked lysosomal storage disease characterized by increased risk of osteoporosis and fractures. The impact of FD on clinical measures of bone quality is unknown. This considered, aim of our study was to evaluate whether trabecular bone microarchitecture, measured by trabecular bone score (TBS), is altered in patients with FD compared to control subjects.

**Methods:**

This retrospective monocentric study enrolled 14 patients (M/F 1/1, median age 46 [37–63] years, range 31–72 years) newly diagnosed with FD between January 2016 and July 2023 who underwent dual-energy X-ray absorptiometry (DXA) image at the time of diagnosis and 42 matched controls. In all subjects, data about bone mineral density (BMD) and lumbar spine TBS were collected and total calcium, parathyroid hormone (PTH), 25(OH) vitamin D, alkaline phosphatase (ALP), creatinine and estimated glomerular filtration rate (eGFR) were evaluated. In subjects with FD, globotriaosylsphingosine (lyso-Gb3), 24-hour proteinuria and albumin-creatinine ratio were also assessed.

**Results:**

Patients with FD presented significantly lower lumbar spine TBS (1.29 [1.22–1.38] vs. 1.42 [1.39–1.47], *p* < 0.001) and lower lumbar spine BMD (0.916 ± 0.166 vs. 1.031 ± 0.125 g/cm^2^, *p* = 0.008) compared to controls; moreover, FD was shown to be an independent risk factor for both low lumbar spine TBS (β = -0.118, *p* < 0.001) and BMD (β = -0.115, *p* = 0.009). No differences were found in serum calcium, ALP, 25(OH) vitamin D and eGFR in both groups, but FD patients had significantly higher PTH levels compared to controls (*p* = 0.016). Finally, 8 patients with FD presented either moderately or severely increased albuminuria and only 2 patients presented normal lyso-Gb3 levels.

**Conclusion:**

Patients affected by FD present significantly lower lumbar spine TBS and BMD compared to controls. Our findings strongly support the importance of carrying out a thorough evaluation of bone status in all patients affected by FD at baseline.

## Introduction

Fabry disease (FD) is a rare X-linked lysosomal storage disease characterized by partial or absolute deficiency of α-galactosidase A (α-Gal A) activity [1]. This enzymatic alteration leads to progressive intracellular accumulation of glycosphingolipids, primarily globotriaosylceramide (Gb3), and globotriaosylsphingosine (lyso-Gb3) in several cell types with progressing tissue damage and organ failure [2]. The incidence of FD varies widely depending on the population considered, but it is now clear that this condition is more common than previously thought, with a significantly higher prevalence in countries with newborn screening [1, 3–5].

In addition to the classic manifestations of the disease with involvement of organs such as the heart, kidneys, central nervous system, gastrointestinal tract and eyes, several pieces of evidence have indeed shown that endocrine dysfunctions and bone damage are often present [1, 6–12]. In particular, the initial detailed documentation of bone manifestations in FD dates back to 2005, wherein a cohort of 23 French males, aged between 16 and 60 years old and newly diagnosed with FD, revealed that 87% exhibited a low bone mass, either osteopenia (11 subjects) or osteoporosis (9 subjects) [6].

In their study, Mersebach et al. [7] evaluated a heterogeneous population of 53 FD patients, both males and females, pre- and post-menopausal, many of them with a variable degree of renal impairment. Two thirds of this population were affected with lumbar and/or femoral osteopenia/osteoporosis and menopause and renal failure were shown to be the main predictors of bone status.

Moreover, a retrospective analysis of bone mineral density (BMD) results and fracture incidence in 44 FD patients (22 males and 22 females) was undertaken by Talbot and colleagues [8]. They showed that a low BMD was highly prevalent in male patients with increased incidence of non-traumatic fractures.

Finally, more recently, a Japanese study group suggests that such a reduction in BMD may even be reversible after initiation of enzyme replacement therapy (ERT), at least in male subjects [11].

In the evaluation of bone health, the measurement of BMD certainly represents a fundamental aspect, as it constitutes the primary method for estimating fracture risk. Indeed, this safe and cost-effective approach to assessing bone mass has been consistently demonstrated to predict fracture risk in epidemiological studies and randomized clinical trials [13, 14].

BMD measurement, however, provides only a partial result, as reduced BMD is known to account for approximately 70% of fragility fractures observed in clinical practice [15]. This probably indicates that other elements may play a relevant role in bone strength and then help to identify patients at high risk for fractures.

In this regard, trabecular bone score (TBS) is a partial novel textural index to evaluate bone microarchitecture based on the lumbar spine of dual-energy X-ray absorptiometry (DXA) image [16] which has proved to be useful in particular in several settings of secondary osteoporosis [17–20]. A higher TBS value indicates improved skeletal texture, reflecting better microarchitecture, while a lower TBS value suggests weaker skeletal texture, indicating degraded microarchitecture [17]: specifically, a value below 1.31 indicates partially degraded microarchitecture with intermediate fracture risk, while a value below 1.23 indicates degraded microarchitecture with high fracture risk [21].

Finally, other methods for qualitative bone assessment include hip Structural Analysis (HAS) and bone strain index (BSI). The former provides a mechanical characterization of three femoral regions of interest (narrow neck, intertrochanteric, and femur shaft) and has demonstrated the ability to predict subsequent hip fracture occurrence in certain studies [22], while the latter is an innovative DXA-derived deformation index, which considers information on bone geometry and resistance to loadings on local areas [23].

As of today, however, there is no definitive data whether the bone deterioration present in FD is limited to a reduction in BMD or it also involves alterations in bone quality. In this regard, the aim of our study was to evaluate whether trabecular bone microarchitecture, measured non-invasively by TBS, is altered in patients with FD compared to control subjects.

## Materials and methods

This retrospective monocentric study was conducted at the Division of Endocrinology, Diabetology and Metabolism of the University Hospital “Città della Salute e della Scienza di Torino” (Turin, Italy). The study group comprised 14 patients (7 males and 7 females, belonging to 11 different families) newly diagnosed with FD between January 2016 and July 2023 who underwent DXA at the time of diagnosis and 42 sex-, age- and body mass index (BMI)-matched controls. The diagnosis of FD was confirmed in all patients by genetic testing (Table 1). Variants were classified according to the American College of Genetics and Genomics (ACMG) guidelines [24].

**Table 1:** Demographic characteristics of patients with Fabry disease (FD) at the time of diagnosis and list of variants responsible for the clinical picture. Variants are classified according to the guidelines provided by the American College of Medical Genetics and Genomics (ACMG). Accordingly, C5 variants are pathogenic, C4 are likely pathogenic and C3 are variants of unknown significance. Subjects 1, 2, 3 and 4 belong to the same family. Lyso-Gb3: globotriaosylsphingosine

Patient	Age at diagnosis	Sex	Mutation	Variants classification	FD Phenotype	Lyso-Gb3 (ng/mL) *n*.v. ≤1.8
1	63	Female	c.65T > G, p.V22G	C3	Full-blown	1.6
2	41	Male	c.65T > G, p.V22G	C3	Full-blown	2.8
3	40	Male	c.65T > G, p.V22G	C3	Full-blown	3.6
4	39	Male	c.65T > G, p.V22G	C3	Full-blown	5.0
5	57	Female	c.552T > G, p.Tyr184	C4	Full-blown	8.5
6	51	Male	c.644 A > G, p.N215S	C4	Prominent cardiac	6.3
7	36	Male	c.427G > A, p.A143T	Conflicting interpretations (most likely a disease modifier)	Peripheral and auditory neuropathy	2.3
8	36	Female	c.979 C > A, p.Q327K	C5	Full-blown	1.7
9	56	Female	c 902G > A, p.R301Q	C5	Full-blown	3.4
10	37	Female	c.729G > C, p.L243F	C4	Full-blown	4.6
11	31	Female	c.424T > C, p.C142R	C5	Full-blown	5.8
12	72	Male	c.247G > A, p.D83N	C4	Full-blown	2.1
13	71	Male	c.644 A > G, p.N215S	C4	Prominent cardiac	2.4
14	68	Female	c.901 C > G, p.R301G	C5	Full-blown	3.2

Information about weight (kg), height (m), BMI (kg/m^2^), smoking habits, previous fractures, drug history and history of diabetes mellitus were collected. Main secondary causes of osteoporosis were excluded in all FD patients; four women with FD were postmenopausal while younger women (*n* = 3) presented regular menses.

The following variables were evaluated in all subjects: total calcium, parathyroid hormone (PTH), 25(OH) vitamin D, alkaline phosphatase (ALP), creatinine and estimated glomerular filtration rate (eGFR, calculated through the CKD EPI [Chronic Kidney Disease Epidemiology Collaboration]); in subjects with FD, lyso-Gb3 levels, as well as 24-hour proteinuria and albumin-creatinine ratio (ACR) were also assessed.

According to current Kidney Disease Improving Global Outcomes (KDIGO) Clinical Practice Guidelines for the Evaluation and Management of Chronic Kidney Disease (CKD), CKD was defined as either eGFR < 60 mL/min/1.73m^2^, presence of moderately or severely increased albuminuria or previous renal transplantation.

Moderately and severely increased albuminuria were defined by ACR between 30 and 300 mg/g and > 300 mg/g, respectively [25]. Proteinuria was defined by excretion of > 150 mg/day of proteins.

The study was approved by the Local Ethics Committee (cod. 0054585) and was in accordance with the principles of the Declaration of Helsinki. Written informed consent was obtained from all study participants.

### Laboratory measurements

PTH levels (pg/mL) were measured by chemiluminescence immunoassay (Diasorin). The sensitivity of the method was 1.7 pg/mL and normal values were 6.5–36.8 pg/mL; intra- and inter-assay coefficient of variation (CV) were 3.8% and 4.3%, respectively.

25(OH) vitamin D values (ng/mL) were assessed through chemiluminescence microparticle immunoassay (Abbott). The sensitivity of the method was 2.1 ng/mL and normal values were > 30 ng/mL; intra- and inter-assay CV were 4.1% and 5.2%, respectively.

Serum calcium (mmol/L) was assessed by a photometric assay (Beckman). The sensitivity of the method was 0.01 mmol/L and normal values were 2.20–2.65 mmol/L; intra- and inter-assay CV were 0.5% and 0.9%, respectively.

Alkaline phosphatase (ALP) serum levels were measured by a photometric assay (Beckman). The sensitivity of the method was 1 UI/L and normal values were 43–115 UI/L for men and 33–98 UI/L for women. Intra- and inter-assay CV were 4% and 7%, respectively.

Lyso-Gb3 levels (ng/mL) were measured using mass spectrometry (LC/MRM-MS) at the CENTOGENE AG (Rostock; Germany). Normal values were ≤ 1.8 ng/mL.

All other biochemical variables were assayed in serum or urine according to the automated methods currently used in the analysis laboratory of our center.

### Measurement of BMD and TBS and assessment of vertebral fractures

Lumbar spine (L1–L4) and femoral BMD measurements were performed using the DXA technique (Hologic densitometer) and the analyzed locations were at the lumbar spine and at the femoral neck and total hip sites. Results were presented as BMD (g/cm^2^), T-score and Z-score.

The diagnosis of osteoporosis and osteopenia were determined, respectively, by a BMD T-score ≤ − 2.5 standard deviations (SD) or between − 1 and − 2.5 SD at any skeletal site in men over 50 years old and in postmenopausal women; in women with premenopausal status or in patients younger than 50 years a Z-score ≤ − 2.0 was regarded as below the expected range for age.

TBS values were obtained from lumbar spine DXA images using TBS iNsight™ software, version 3.0.2.0 (Med-Imaps, Pessac, France). Considering that the role of TBS in bone health assessment has not yet been validated in subjects with a BMI below 15 or above 37 kg/m^2^, only individuals with a BMI within these two values were considered for the analysis.

Finally, all patients with FD underwent vertebral morphometry imaging performed by DXA in order to detect vertebral fractures. Vertebral fractures were defined as reductions of at least 20% in one vertebral body’s height according to Genant’s criteria.

### Statistical analysis

Normally and non-normally distributed variables were expressed as mean and SD or median and interquartile range (IQR), respectively, while categorical data were expressed as counts and percentages. Normality was assessed using the Shapiro-Wilk test.

Differences between groups were evaluated by Student’s t test for independent samples in the case of variables with normal distribution; to highlight the differences between the median values of non-normally distributed variables Mann-Whitney test was used when appropriate.

The chi-square test and the Fisher’s exact test were used to evaluate the association between binary variables, while the Spearman’s test was used to evaluate the correlation of continuous ones. Additionally, simple linear and stepwise multiple regression analysis was performed to identify the predictors of TBS and BMD values.

A cut-off of p value < 0.05 was considered as statistically significant. Statistical analysis was performed using MedCalc^®^ (Statistical Software version 20.007, MedCalc Software Ltd, Ostend, Belgium). Figures were made using GraphPad Prism (version 8.0.2; GraphPad Software Inc., La Jolla, California).

## Results

### Patient characteristics

The general characteristics of patients with FD and controls are presented in Table 2. Two patients in the FD group and 3 controls were affected by diabetes mellitus and they were all taking oral anti-diabetic drugs.

**Table 2:** General characteristics of patients with Fabry disease and controls. Data are expressed as mean ± standard deviation (SD) or median and interquartile range (IQR) or n (%). *BMI* body mass index, *PTH* parathyroid hormone, *ALP* alkaline phosphatase, *eGFR* estimated glomerular filtration rate, *ACR* albumin-creatinine ratio, *Lyso-Gb3* globotriaosylsphingosine, *BMD* bone mineral density, *TBS* trabecular bone score

	Overall (*n* = 56)	Fabry disease (*n* = 14)	Controls (*n* = 42)	*p*-value
Age *(years)*	50 (38.5–62)	46 (37–63)	51 (39–62)	0.857
Sex, male, *n (%)*	28 (50)	7 (50)	21 (50)	1.000
BMI *(kg/m*^*2*^*)*	25.0 (23.0-26.3)	23.5 (21.3–25.6)	25.6 (23.9–26.4)	0.054
Diabetes mellitus, *n (%)*	5 (8.9)	2 (14.3)	3 (7.1)	0.589
Smoking habit, *n (%)*	16 (28.6)	4 (28.6)	12 (28.6)	1.000
Serum calcium, mmol/L [normal values 2.2–2.65]	2.38 (2.30–2.44)	2.33 (2.26–2.44)	2.38 (2.35–2.44)	0.168
25(OH) vitamin D, ng/mL [normal values > 30]	20.8 (15.4–27.6)	17.9 (13.4–23.9)	20.8 (16.9–29.0)	0.158
PTH, pg/mL [normal values 6.5–36.8]	20.9 (17.7–28.0)	29.9 (19.5–40.8)	20.1 (16.1–26.6)	**0.016**
ALP, UI/L [normal values 33–98 in women, 43–115 in men]	69 (60–85)	70 (62–89)	67 (59–83)	0.656
Creatinine, mg/dL [normal values 0.55–1.02]	0.89 (0.74-1.00)	0.81 (0.65–0.95)	0.90 (0.80-1.00)	0.186
eGFR, mL/min [calculated with the formula of Chronic Kidney Disease Epidemiology Collaboration, CKD-EPI]	96 (81–105)	99 (85–107)	94 (80–100)	0.343
ACR, mg/g [normal values < 30]		33.1 (7.0-60.1)		
24-h proteinuria, mg/day [normal values < 150]		142 (98–472)		
Lyso-Gb3, ng/mL [normal values ≤ 1.8]		3.8 ± 2.0		
Osteopenia, *n (%)*	12 (21.4)	3 (21.4)	9 (21.4)	1.000
Osteoporosis, *n (%)*	4 (7.1)	2 (14.3)	2 (4.8)	0.258
BMD below the expected range for age, *n (%)*	3 (5.4)	3 (21.4)	0 (0)	**0.013**
TBS	1.40 (1.35–1.46)	1.29 (1.22–1.38)	1.42 (1.39–1.47)	**< 0.001**
Lumbar spine BMD (g/cm²)	1.002 ± 0.144	0.916 ± 0.166	1.031 ± 0.125	**0.008**
Lumbar spine T-score	-0.3 (-1.1-[0.5])	-1.3 (-2.7-[-0.3])	-0.1 (-0.9-[0.6])	**0.006**
Lumbar spine Z-score	-0.2 (-0.8-[0.4])	-0.7 (-2.6-[0.1])	-0.1 (-0.5-[0.6])	**0.046**
Femoral neck BMD (g/cm²)	0.790 ± 0.134	0.742 ± 0.128	0.803 ± 0.134	0.180
Total hip BMD (g/cm²)	0.932 ± 0.143	0.885 ± 0.149	0.945 ± 0.140	0.222
Femoral neck T-score	-0.9 (-1.9-[-0.4])	-1.4 (-2.2-[-0.8])	-0.8 (-1.8-[-0.3])	0.142
Femoral neck Z-score	-0.3 (-0.7-[0.3])	-0.5 (-0.9-[0.2])	-0.2 (-0.5-[0.4])	0.340
Total hip T-score	-0.3 (-1.4-[0])	-0.6 (-1.5-[-0.3])	-0.3 (-1.4-[0])	0.222
Total hip Z-score	0 (-0.7-[0.4])	-0.5 (-1-[0.6])	0.1 (-0.4-[0.3])	0.461

No patients reported history of previous fractures nor were any new vertebral fractures detected at vertebral morphometry. None of the patients with FD had ever taken anti-osteoporosis medications and only two patients were already on vitamin D supplementation treatment at the time of FD diagnosis. One woman had undergone liver and kidney transplantation 12 years before the diagnosis of FD for previous hepatorenal polycystosis and had been on low-dose glucocorticoid therapy since then.

No differences were found in serum calcium, 25(OH) vitamin D, ALP and creatinine in both groups, but FD patients had significantly higher PTH levels compared to controls (*p* = 0.016); moreover, 5 patients with FD (35.7%) and 3 controls (7.1%) presented secondary hyperparathyroidism (*p* = 0.015). 100% of patients with FD and 31/42 controls (73.8%) had vitamin D insufficiency (i.e. <30 ng/mL [26], *p* = 0.034); however, no difference between the two groups (8/14 [57.1%] vs. 15/42 [35.7%]) was observed regarding vitamin D deficiency (i.e. <20 ng/mL [26], *p* = 0.162).

At the bivariate analysis PTH levels were negatively correlated with 25(OH) vitamin D in controls (*r* = -0.393, *p* = 0.012) while just a trend was observed in subjects with FD (*r* = -0.479, *p* = 0.098); on the other hand, no correlation was appreciated between PTH values and ALP, creatinine, eGFR, ACR and 24-h proteinuria.

According to ACR values, 4 patients were classified as having moderately increased albuminuria and 4 as having severely increased albuminuria; finally, 7 subjects presented a 24-h proteinuria > 150 mg/day (range 151–872 mg/day). As a consequence, only 6/14 subjects with FD (42.9%) did not present any kidney impairment.

Considering lyso-Gb3 levels, only 2 female patients presented normal values (mean 3.8 ± 2.0 ng/mL; range 1.6–8.5 ng/mL) (Table 1).

### Comparison of TBS between patients with FD and control subjects

**Fig. 1:**
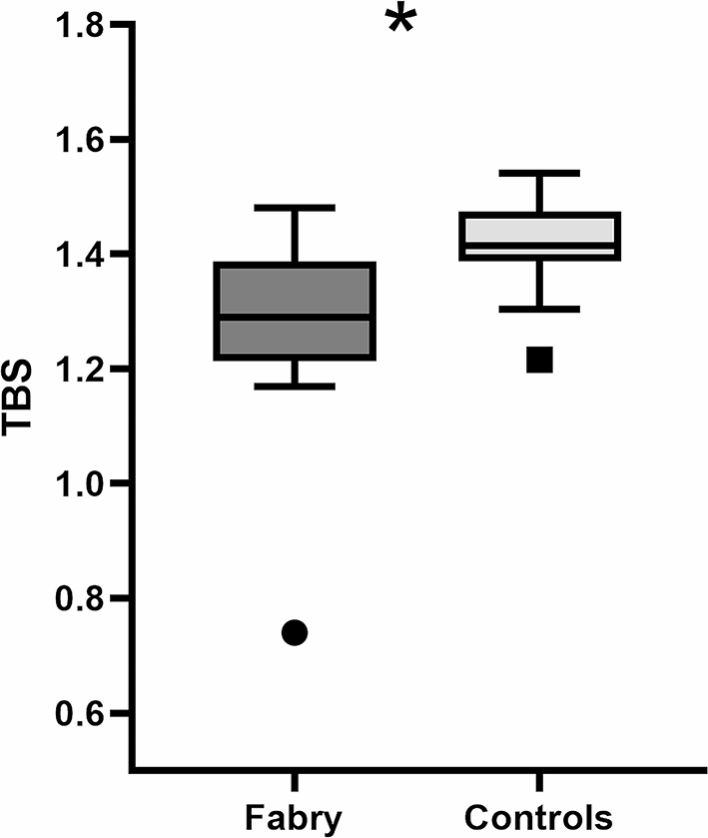
Trabecular bone score (TBS) in patients with Fabry disease and controls. *p < 0.001

Patients with FD presented significantly lower lumbar spine TBS compared to controls (1.29 [1.22–1.38] vs. 1.42 [1.39–1.47], *p* < 0.001) (Fig. 1) and this was confirmed even after removing from the analysis patients with CKD and their respective controls (1.24 [1.20–1.36] vs. 1.41 [1.38–1.46], *p* = 0.019).

At the bivariate analysis TBS was negatively correlated with age in controls (*r* = -0.309, *p* = 0.047) but not in subjects with FD (*r* = -0.052, *p* = 0.859); conversely, only in individuals with FD, TBS correlated negatively with ACR (*r* = -0.541, *p* = 0.046) and PTH levels (*r* = -0.600, *p* = 0.030) and positively with 25(OH) vitamin D levels (*r* = 0.586, *p* = 0.028). No correlations were evidenced between TBS and either lyso-Gb3, serum calcium, ALP, creatinine, eGFR and 24-h proteinuria.

Finally, TBS was significantly correlated with lumbar spine BMD in controls (*r* = 0.411, *p* = 0.007) while just a trend was observed in subjects with FD (*r* = 0.523, *p* = 0.055); likewise, a negative correlation was noted between TBS and BMI in individuals affected by FD (*r* = -0.710, *p* = 0.005) whereas just a trend was observed in controls (*r* = -0.301, *p* = 0.053).

A stepwise multivariate correlation analysis was then performed in order to identify parameters that could independently predict TBS value. TBS significantly correlated with FD (β = -0.118, *p* < 0.001), 25(OH) vitamin D (β = 0.038 per 10 ng/mL increase, *p* = 0.003), lumbar spine BMD (β = 0.341, *p* < 0.001) and BMI (β = -0.018, *p* < 0.001), together explaining 70% of the variance in TBS values.

### Comparison of BMD between patients with FD and control subjects

No differences regarding the proportion of subjects with osteopenia or osteoporosis were reported in the two groups but FD was significantly associated with the presence of BMD below the expected range for age (*p* = 0.013).

**Fig. 2:**
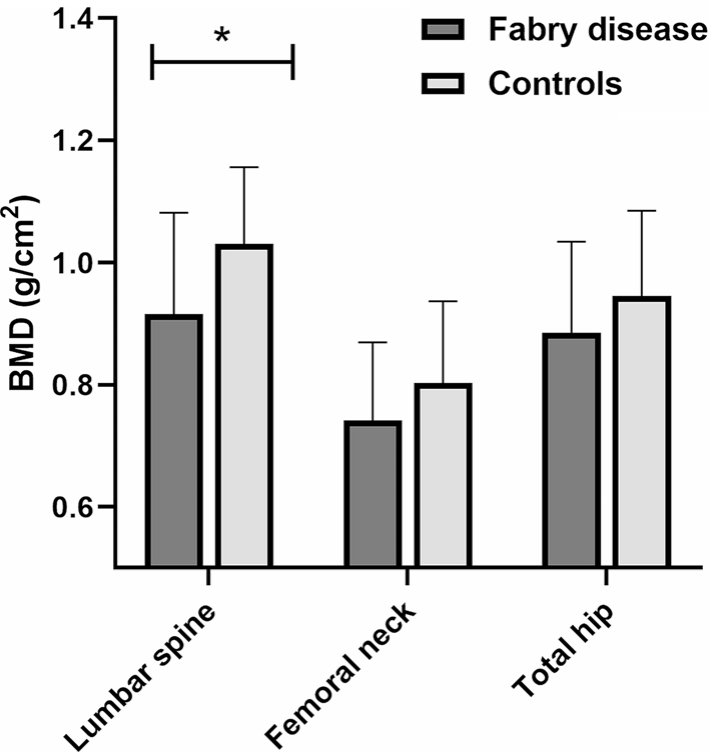
Comparison of bone mineral density (BMD) between patients with Fabry disease and controls. *p = 0.008

Patients with FD presented significantly lower lumbar spine BMD compared to controls (0.916 ± 0.166 vs. 1.031 ± 0.125, *p* = 0.008) while no differences were observed regarding BMD at femoral neck or total hip (Fig. 2): as a result, both T-score and Z-score measured at the lumbar spine were significantly lower in patients affected by FD (*p* = 0.006 for T-score, *p* = 0.046 for Z score).

At the bivariate analysis femoral neck and total hip BMD were negatively correlated with age (*r* = -0.508, *p* < 0.001 at femoral neck, *r* = -0.371, *p* = 0.007 at total hip) while no association was observed at the lumbar spine (*r* = -0.068, *p* = 0.617). Moreover, FD was negatively associated with lumbar spine BMD (β = -0.115, *p* = 0.008) and this was confirmed at the multivariate analysis even after accounting for age (β = -0.115, *p* = 0.009). Eventually, no correlation was found between lyso-Gb3 levels and BMD at any skeletal site.

## Discussion

To the best of our knowledge, this is the first study to investigate the potential involvement of microarchitectural bone deterioration in individuals affected by FD using TBS. Specifically, our findings demonstrate that subjects with FD display not only a simple reduction in BMD, but also qualitative alterations in bone tissue, indicating broader bone damage; moreover, FD was an independent predictor of low lumbar spine TBS and BMD at bivariate and multivariate analyses.

Bone involvement in patients with FD has long been misrecognized and underestimated and only recently it has gained significant attention. In this regard and in order to possibly mitigate the increased risk of osteoporosis, current consensus recommendations suggest early assessment of vitamin D status starting as early as childhood [2].

Our data confirm that bone is rarely spared in patients with FD, and in our population only 6/14 individuals had a normal bone picture.

However, the most notable feature is that bone involvement appears to be particularly early, given that as many as 3 subjects among those younger than 50 years old (3/7) exhibited a BMD below the expected range for age; on the other hand, the proportion of subjects with osteoporosis and osteopenia was similar between the two groups.

Furthermore, as already reported by some [6, 11] but not all studies [7, 8], bone deterioration seems to be particularly evident at the lumbar spine. Our results are in line with this: indeed, considering the 3 young subjects who were diagnosed with “BMD lower than expected for age”, the sole assessment of the Z-score at the femoral neck or total hip would have led to a misdiagnosis of “BMD within the expected range for age”.

Several possible causes of osteopenia and osteoporosis in FD patients have been proposed. The use of antiepileptic drugs such as carbamazepine is one possible cause of bone loss [8] but no patients in our population were taking them.

Moreover, unlike other studies [7], in our population we did not find a correlation between BMD and renal function. In our cohort, however, eGFR was preserved in all patients, including the woman who had previously undergone renal transplantation, while kidney impairment was mainly represented by persistent albuminuria, present in more than half of the subjects.

Vitamin D insufficiency is highly prevalent among individuals with FD, often due to either limited sun exposure, CKD or intestinal malabsorption [27]. Certainly, heat intolerance is a common complication, frequently attributed to concurrent neuropathy, with an incidence in FD patients ranging up to almost 40% of subjects, potentially elucidating this phenomenon [2, 28]. Similarly, patients with FD may present underlying cardiac disorders that can impair physical exercise and, conversely, facilitate home staying [29]. Finally, the accumulation of Gb3 deposits in the skin has been proposed as a possible interference in vitamin D synthesis [29, 30]. Indeed, compared to controls, subjects with FD were significantly more likely to have 25(OH) vitamin D levels consistent with insufficiency [26], even though two of them were already receiving cholecalciferol supplementation.

FD patients had higher PTH levels than controls, although there were no significant differences in serum calcium, 25(OH) vitamin D and eGFR values; however, it is relevant to note that 11 controls were already taking cholecalciferol supplementation at the time of the evaluation.

Even though statistical significance was not reached in our cohort, it has already been reported in the literature how PTH levels can potentially increase in proteinuria, thus reflecting the progression of tubular damage [31]. Moreover, FD frequently affects the gastrointestinal tract and it has been previously proposed as a potential contributor to the increased risk of malabsorption, which is another well-known cause of secondary hyperparathyroidism [32, 33]. Daily calcium intake can be reliably inferred through 24-hour urinary calcium excretion, but such measurement was not available in our study. Therefore, neither hypo- nor hypercalciuria could be ruled out in our FD population [33, 34].

Despite a higher prevalence of secondary hyperparathyroidism, patients with FD appear to demonstrate greater involvement of trabecular rather than cortical bone. All this considered, it is plausible that the underlying mechanisms of the characteristic bone damage in FD are closely intrinsic to the disease itself. Additionally, no differences in ALP levels were observed between the two groups. Even if data regarding bone-specific alkaline phosphatase (BAP) as well as key markers of bone resorption (such as serum C-terminal telopeptide [CTX]) were not available, this finding may support a rather impaired bone formation in patients affected by FD.

Finally, BMD was negatively correlated with age at all sites except at the lumbar spine: this should not be surprising, as there is ample evidence that spinal degeneration can appear with age, including vertebral deformities and osteophytes, making this measurement less reliable in particular after age 65 [35].

In any case, no data regarding bone damage at the microarchitectural level using TBS in patients with FD were available to date.

As of today, only one other study [36] has evaluated bone quality in patients with several disorders of inborn errors of metabolism (including FD) using the high-resolution peripheral quantitative computed tomography (HR-pQCT) technique. This is an in vivo bone imaging modality that conducts scans of the distal limbs (radius and tibia) and allows for assessment of volumetric bone density and microarchitecture of cortical and trabecular compartments.

In their work, Sidhu et al. [36] reported a tendency towards cortical and trabecular alteration, although less pronounced compared to other genetic conditions primarily affecting bone; however, the analysis was not focused on FD and it was not clear if patients were evaluated upon diagnosis or they had previously been treated with ERT.

Indeed, our study showed that these patients have significantly lower TBS values than controls and that FD itself is an independent risk factor at multivariate analysis associated with bone microarchitecture deterioration.

Due to its accessibility and simplicity, TBS has become one of the most commonly employed non-invasive methods for assessing bone quality in recent years [37]. Moreover, unlike lumbar spine BMD, TBS is significantly less affected by age-related degenerative changes [38, 39], rendering it a suitable tool for the evaluation of trabecular bone microarchitecture even in the elderly population.

Previous studies in postmenopausal women have already shown that TBS is able to predict the occurrence of fragility fractures regardless of BMD [40, 41]. Furthermore, the evaluation of TBS assumes particular importance in instances of secondary osteoporosis, in which increased fracture risk is often associated with changes in microarchitecture and bone quality rather than depending on reduced BMD [17].

At the bivariate analysis TBS was inversely correlated with PTH and ACR in patients with FD. Data about microarchitecture bone alteration in CKD have already been reported [42, 43], but mainly in individuals with reduced eGFR, while data about moderately and severely increased albuminuria and/or proteinuria are scanty. Anyway, TBS values in patients with FD were shown to be significantly reduced compared to controls even after removing from the analysis patients with CKD, thus limiting the involvement of this comorbidity.

At the multivariate analysis, indeed, TBS was shown to be positively correlated with 25(OH) vitamin D levels and lumbar spine BMD and negatively correlated with BMI. This was not an unexpected finding, as a positive correlation with both 25(OH) vitamin D levels and BMD had already been shown [44, 45]. Likewise, inverse relationship between TBS and BMI has already been reported in the literature [46]: in this regard obesity may have a negative impact on bone quality potentially due to chronic low-grade inflammation [47].

Despite patient matching, a nearly significant trend of lower BMI values in patients with FD compared to controls was observed in our cohort. As previously reported, in fact, FD often involves the gastrointestinal tract and can lead to reduced BMI as a consequence of increased risk of malnutrition [32]. Considering the small sample size of our study, it is even possible that the higher BMI evident in the control group may have partly “reduced” the evidence of degraded TBS in subjects with FD, given the correlation just mentioned [44, 46].

Of note, no correlation between lyso-Gb3 levels at diagnosis and either TBS or BMD was observed, in contrast to the findings of a recent Japanese study which suggested a possible correlation with BMD at the lumbar and femoral neck sites, at least in males [11]. On the other hand, the evidence regarding lyso-Gb3 as a measure of clinical severity and as an indicator of the degree of target organ involvement with prognostic significance, still requires further investigation [48]. Therefore, our present data do not support the role of lyso-Gb3 in this context, at least regarding bone health status.

Our study presents some limitations. Firstly, the population was rather small, and no fractures were detected in patients with FD: this considered, it was not possible to evaluate the potential correlation between TBS and BMD values and the fracture risk, nor to perform sub analyses in subjects stratified for BMD categories. In addition, data on major markers of bone formation (such as BAP) as well as markers of bone resorption (such as CTX) were not available, although no differences were evidenced regarding ALP levels.

Finally, only data at baseline at the time of FD diagnosis were evaluated, so any potential beneficial effect of ERT or chaperone treatment on TBS and long-term bone quality, although intriguing, remains yet fully to be demonstrated. In fact, the paper by Nose and colleagues which suggested an improvement in BMD correlated with lyso-Gb3 reduction following the initiation of ERT [11] is severely limited in its actual clinical meaning, considering the very small sample of patients. On the other hand, the recent study by Aitken et al. showed that subjects with FD treated with medical therapy had worse bone density trajectories compared to those who were not receiving any specific treatment. This data suggests that Fabry-specific therapies may not be able to reverse all disease mechanisms, such as those responsible for the underlying bone damage [12].

## Conclusion

The results of the present study show for the first time that patients affected by FD not only present a reduced BMD but also a significant impairment of bone microarchitectural characteristics, as documented by lower lumbar spine TBS compared to controls. Moreover, FD itself was shown to be an independent predictor of low lumbar spine TBS and BMD at bivariate and multivariate analyses.

From a clinical point of view, also considering the frequent finding of either vitamin D deficiency or insufficiency, as well as the presence of risk factors and/or secondary causes of osteoporosis in FD, our findings strongly support the importance of carrying out a thorough evaluation of bone status in all patients affected by FD at baseline.

## Data Availability

The data sets generated during and/or analyzed during the current study are not publicly available but are available from the corresponding author on reasonable request.
